# NMR-Derived Salt Bridges in Insulin Analogue: Resolving Artifactual Overbinding in Molecular Dynamics via Charge Scaling

**DOI:** 10.1021/acs.jpclett.5c01786

**Published:** 2025-07-15

**Authors:** Ngoc Lan Le Nguyen, Jiří Žák, Pavel Jungwirth, Martin Lepšík

**Affiliations:** Institute of Organic Chemistry and Biochemistry of the Czech Academy of Sciences, Flemingovo nám. 542, 160 00 Prague 6, Czech Republic

## Abstract

Salt bridges are ionic interactions that are of great importance in protein recognition. However, their structural description using X-ray crystallography or NMR may be inconclusive. Classical molecular dynamics (MD) used for the interpretation neglects electronic polarization, which results in artifactual overbinding. Here, we resolve the problem via charge scaling, which accounts for electronic polarization in a mean-field way. We study three salt bridges in insulin analogue. New NMR ensembles are generated via NOE-restrained MD using ff19SB and CHARMM36m force fields and the scaled-charge prosECCo75. Tens of μs of unrestrained MD show in a statistically converged manner that ff19SB induces a non-native salt bridge. This behavior is quantified via umbrella sampling of salt bridge dissociation, which indicates a rather high strength of up to 4 and 5 kcal mol^–1^ for CHARMM36m and ff19SB, respectively. In contrast, prosECCo75 gives a biologically reasonable dissociation barrier of 1 kcal mol^–1^. Our results indicate that a physically justified description of charge–charge interactions within a nonpolarizable MD framework reliably describes aqueous biomolecular systems.

## Graphical abstract

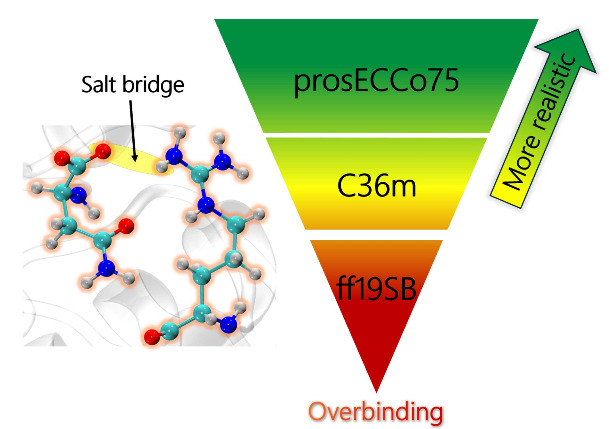

Charge–charge interactions are among the fundamental physical phenomena in chemical processes, playing crucial roles in molecular structure and recognition. Their correct description in MD simulations is essential for accurate modeling of chemical and biological systems and thus advancing material science as well as computer-aided drug design.^1–3^ The commonly used nonpolarizable force fields, however, tend to exaggerate charge–charge interactions due to the absence of additional attenuation caused by electronic polarization. To address this limitation without additional computational costs, the charge scaling method,^4,5^ introduces electronic polarization effects through a mean-field approach denoted as the Electronic Continuum Correction (ECC).^6^ During the past decade, ECC-based models have been extensively developed and applied to various systems, including ionic solutions,^7–16^ protein–ion interactions,^17–20^ lipids,^21–23^ solid surfaces and their interfaces with aqueous solutions,^24–27^ biological systems,^28–30^ and ionic liquids.^31–33^ Overall, the use of ECC qualitatively improved the results, often aligning the computational predictions quantitatively with the experimental results.

Salt bridges are ubiquitous ion–ion interactions that determine the shapes of biomolecules, their specific recognition, and thus underlie many important processes in molecular biology.^34–37^ In proteins, they occur between acidic carboxylates of Glu or Asp side chains or the C-termini and basic Lys, Arg, or His­(+) side chains or the N-termini within a cutoff N···O distance of about 3.5 Å. Despite the critical role of salt bridges in biomolecular stability and function, systematic studies quantifying their strength remain limited, with only a few investigations reported in both experimental and computational contexts. The stability of salt bridges is dependent on not only the strength of the noncovalent interaction but also its solvent exposure, hydrophilicity of the surroundings, and conformational entropy. Thus, the two charged groups can form direct contact pairs or solvent-shared pairs or become completely separated. Salt bridges become typically stronger, with stabilization free energies of about 3–5 kcal mol^–1^, when partially buried inside proteins or shielded from the protein surface due to reduced dielectric effects,^38,39^ while surface-exposed ones are disfavored by increased solvation and conformational entropy, with their strength ranging from 0.2–0.5 kcal mol^–1^.^40–42^ A theoretical study using molecular dynamics simulations with the OPLS-AA force field on a capped Lys–Glu dipeptide in water clusters supports this view. The results show that salt bridge strength decreases with increasing hydration—from approximately 3.7 kcal mol^–1^ in a 20-water molecule cluster to around 2.2 kcal mol^–1^ in a 150-water molecule cluster—highlighting the role of solvent screening in modulating electrostatic interactions.^43^ To extrapolate to the bulk solvent, a smaller model, namely, the NH_4_^+^ ··· HCOO^–^ ion pair, was calculated, yielding nearly identical results (approximately 3.9 kcal mol^–1^ in a 20-water molecule cluster and 2.4 kcal mol^–1^ in the bulk water). In addition, a comprehensive study of salt bridge interactions across various force fields, including AMBER, CHARMM, and OPLS, was conducted using MD simulations of amino acid analogues (Arg/Asp, Lys/Asp, and His­(+)/Asp) in an explicit solvent.^44^ One Arg/Asp capped amino acid dipeptide salt bridge was also simulated. Given the availability of experimentally measured association constant values of the oppositely charged pairs, the simulations revealed that most of the tested force fields overestimated the strength of the salt bridges. The free energies obtained from potential of mean force calculations are about 2–3 kcal mol^–1^ for the amino acid analogues and about 2–4 kcal mol^–1^ for the capped amino acid dipeptide. Overall, previous scarce molecular simulation studies have primarily focused on salt bridge stability of simplified systems such as amino acid analogues or capped dipeptides. An insight into salt bridge behavior at the molecular level within complex and biologically relevant protein environments is still lacking.

To address this issue, we have chosen a structurally very well characterized and relatively small system—insulin and its analogues.^45^ These peptides consist of two interconnected chains, A and B, which make up, in total, about 50 amino acids. The three possible salt bridges are inferred from the available high-resolution X-ray crystal structures as follows: the N-terminal primary amino group of A1 and the Glu A4 side chain (A1–A4), the C-terminal carboxylate of A21 and the Arg B22 side chain (A21–B22), and the side chains of Glu A17 and Arg B22 (A17–B22). A refined experimental reference is derived from the existing NOE distance restraints.^46^ They serve for restrained MD simulations using the standard ff19SB^47^ and CHARMM36m (C36m),^48^ alongside the recent scaled-charge prosECCo75 ^49^ protein force fields, to yield extended NMR ensembles. These, in turn, are compared to unrestrained MD performed with the same parameter sets. Further, to quantify the Gibbs free energy required to transition from a contact to a solvent-shared ion pair, umbrella sampling simulations for the two stable salt bridges are carried out.

Four high-resolution crystallographic structures of human insulin and its analogues (resolution of 1 Å or better) were examined for the presence of salt bridges. In the order of decreasing prevalence in all the chains and alternative conformations, A1–A4 was found in 8 cases of occurrences, A21–B22 had 5 occurrences, and A17–B22 only three (Figure 1 and ). Note that in none of the cases, all three salt bridges would be found present at the same time. To go beyond the static view of the frozen crystals, the 30 NMR models of the l-HisB24 analogue (PDB 2M2N^46^) were analyzed. The occurrences of the three salt bridges were 23.3%, 60.0%, and 0%, respectively. The discrepancy between these orderings may come from different experimental conditions, but also from the specific force field (AMBER) used for the interpretation of the NMR data.^46^

**Figure 1:**
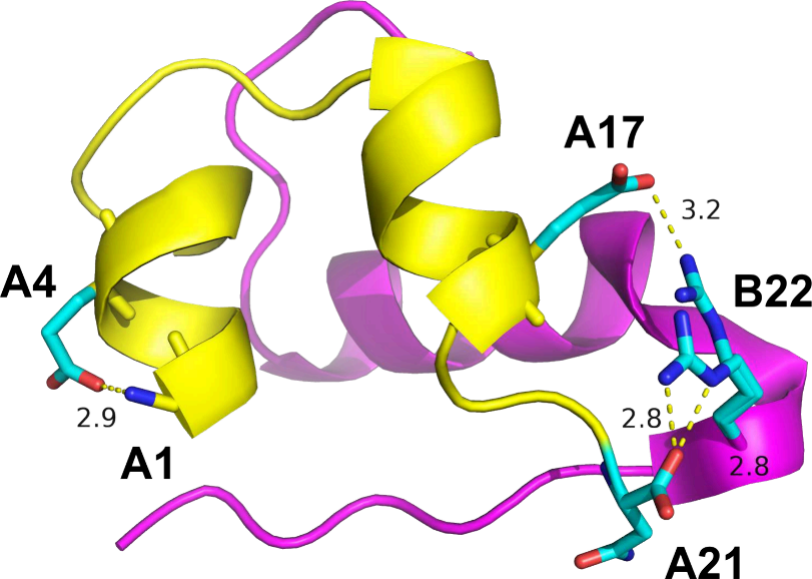
Three possible salt bridges in the insulin analogue: A1–A4, A21–B22, and A17–B22 (PDB 5HQI).^50^ The A and B chains are shown as yellow and magenta cartoon, respectively. The salt bridge residues are shown as sticks with carbon in cyan, oxygen in red, and nitrogen in blue. Distances are given in Å. The figure was prepared using open-source PyMol, version 2.5.^51^

To obtain unbiased and expanded NMR ensembles for reference, four replicas of 100 ns NOE-restrained MD simulations were conducted using the three protein force fields, combined with the standard TIP3P^52,53^ or SPC/E^54^ water models. The stability of the secondary structures was maintained in all of the setups with an average root-mean-square deviation (RMSD) values of 0.6–0.7 Å (). In comparison, four replicas of 10 μs of unrestrained MD simulations gave average RMSDs of the secondary structure backbone twice larger but still reasonably small, about 1.2 to 1.4 Å (). The time evolution of RMSDs suggests that some equilibration occurred in the first 2.5 μs (). Therefore, only the salt bridge occurrences in the last 7.5 μs were taken into account for further analyses.

Figure 2 shows the salt bridge occupancies extracted from all of the MD simulations using the TIP3P water model. Very similar trends were observed for the SPC/E water model (), with some quantitative differences noted below. For the A1–A4 and A21–B22 salt bridges, the occupancies from the NOE-restrained MDs are similar (within 23%) to the unrestrained MD (Figures 2ab, ). Also, compared to ff19SB and C36m, prosECCo75 always yields lower occupancies, which is understandable given its scaled charges (). While the trends were maintained, there was a quantitative difference for the A21–B22 case using the SPC/E water model, where the drop in occupancy from ff19SB and C36m to prosECCo75 was reduced from about 50% to 20% ().

**Figure 2:**
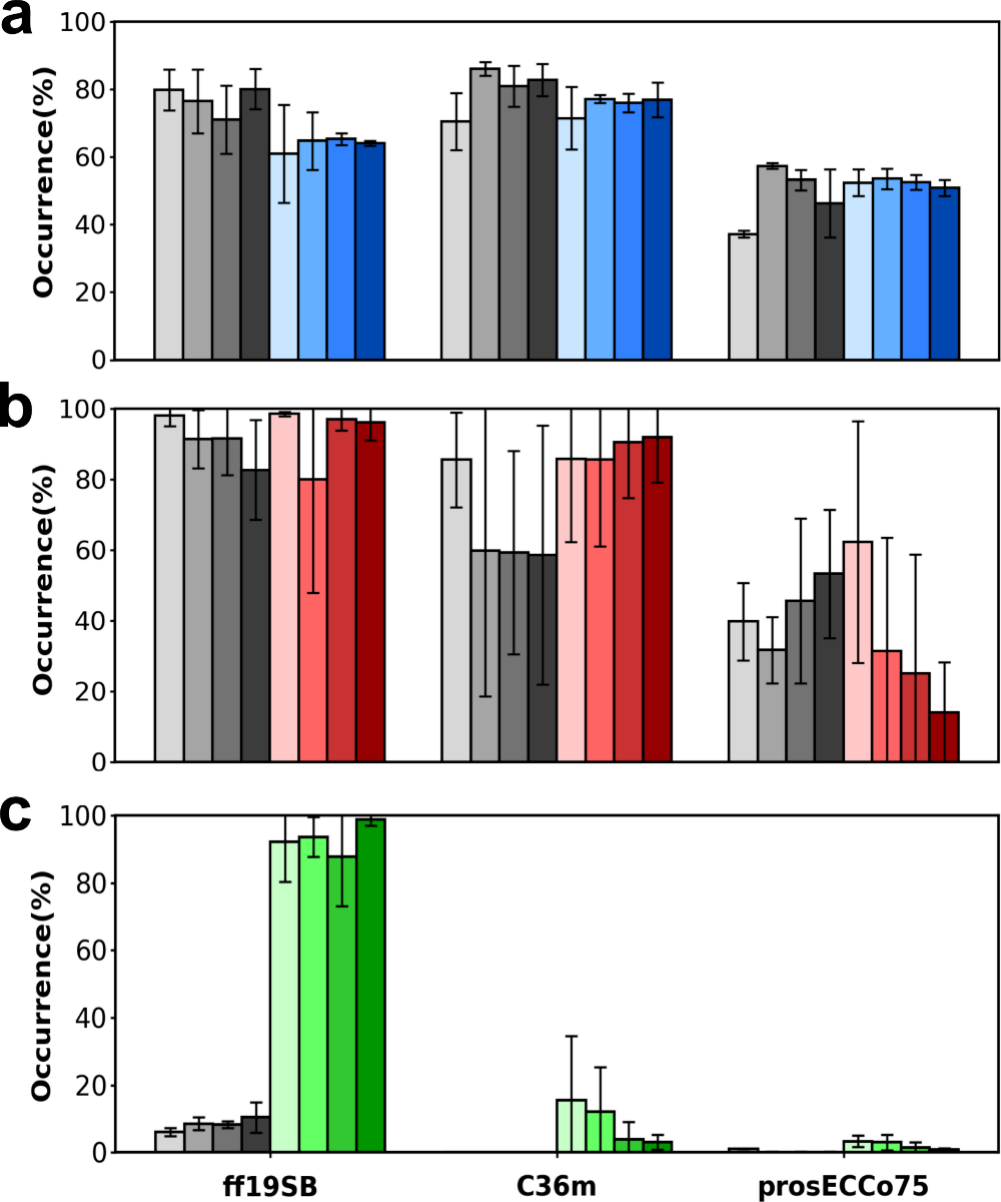
Salt bridge occurrences in MD simulations using the TIP3P water model. NOE-restrained MDs are in gray and unrestrained MDs are colored blue, red, and green, respectively, for a) A1–A4, b) A21–B22, and c) A17–B22. Note that the NOE-restrained occurrences are nonexistent for C36m and prosECCo75 for A17–B22. Each trajectory was divided into four segments of equal length. The height of the bars are averages of the four replicas, and the error bars are the standard deviations.

At a closer inspection of the convergence of these two salt bridges in time, based on comparing the salt bridge occurrences for the individual segments of the production runs, we typically find differences up to 10%, with a few cases reaching 20% (). The differences between the four segments are much smaller in the case of A1–A4 than A21–B22 (Figures 2ab). This is caused by their different locations within the insulin analogue. The N-terminal A1 and Glu A4 form the stable α-helix1 of the A-chain. In contrast, the C-terminal A21–Arg B22 salt bridge is surface-exposed and flexible, hence the larger standard deviations.

The striking case is the A17–B22 salt bridge which, in the NOE-restrained MD, is very weak using ff19SB (8%) and practically nonexistent in NOE-restrained MD using C36m and prosECCo75 (Figure 2c, ). Consistently, unrestrained MD with prosECCo75 shows practically no salt bridge formation and with C36m only a very weak one. In sharp contrast, in unrestrained MD, ff19SB predicts this non-native salt bridge at 80–90% occurrence in both water models. This overbinding and related formation of artifactual charged clusters (Glu A17···Arg B22···Asn A21 C-terminus in this case; Figure 1) calls for caution when using ff19SB for MD or NMR structural interpretation of interactions in proteins involving charged residues.

To inspect the salt bridge strength in more detail, we carried out umbrella sampling simulations to calculate the free energy of dissociation (meaning the transition from the direct contact to the solvent-shared pair) of the two well-defined salt bridges A1-A4 and A21–B22. We note that in agreement with Figure 2c, A17 frequently made contact with B22 during the simulations using ff19SB but it did not using C36m or prosECCo75. The resulting potentials of mean force (PMF) profiles are presented in Figures 3 and for the TIP3P and SPC/E water models, respectively. The estimated boundaries separating the direct contact and solvent-shared conformations of the salt bridges are shown in . The results described below are for the TIP3P water model, but comparable tendencies are obtained using the SPC/E water model as well; see . For the A1–A4 pair, the free energy profiles computed from ff19SB and C36m force fields closely resemble each other (Figure 3, upper left panel) with a transition barrier of 2.5–2.9 kcal mol^–1^. prosECCo75, on the other hand, yields a barrier of half the height, roughly 1.3 kcal mol^–1^. Similarly, the A21–B22 pair exhibits high ion pair stabilities using ff19SB and C36m with free energy barriers of approximately 4.9 and 3.1 kcal mol^–1^, respectively (Figure 3, upper right panel). In contrast, prosECCo75, again in this case, yields the barrier height of one-third or one-half, respectively, with a value around 1.4 kcal mol^–1^. Consistently with previous studies on salt bridge strength, the full-charge ff19SB and C36m force fields tend to overbind, whereas prosECCo75 provides a more realistic stabilization.^40–44,55^

**Figure 3:**
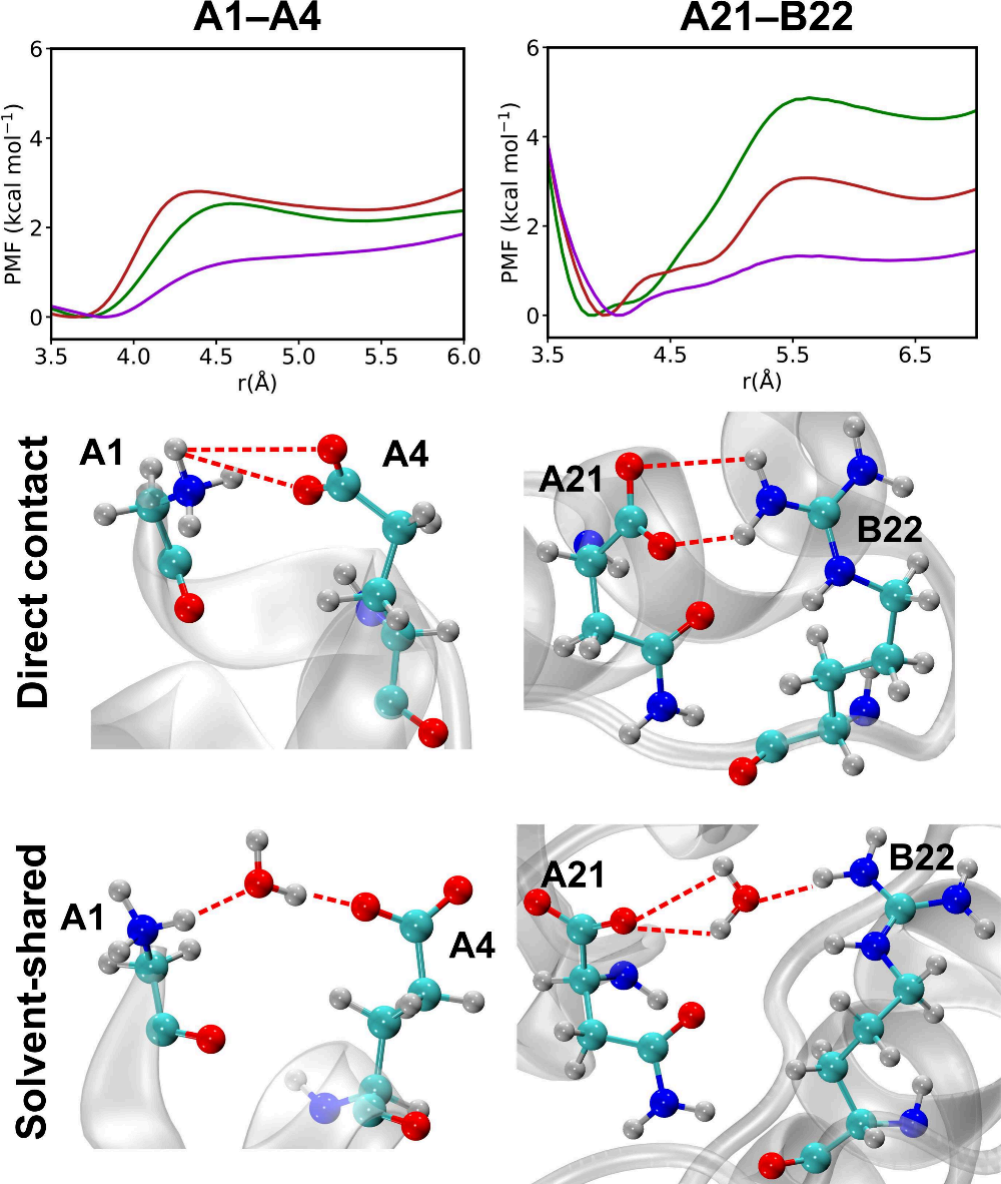
Free energy profiles of salt bridge dissociation for A1–A4 (*r* = *r*_CD‑N_) and A21–B22 (*r* = *r*_C‑CZ_) (obtained from umbrella sampling using ff19SB (green), C36m (red), and prosECCo75 (purple)) are illustrated in the upper panel. The two lower panels show the representative snapshots of direct contact and solvent-shared ion pairs.

Taken together, the description of salt bridges in proteins by standard and scaled-charge force fields was tested in insulin analogues against newly extended NMR ensembles. The standard force fields ff19SB showed a tendency for overbinding in an Arg–Glu salt bridge, thus forming a non-native contact and a charged cluster, observed neither in X-ray nor in the extended NMR ensemble. This behavior was caused by the overpolarization of fixed partial charges.

A simple, yet physically well grounded solution with no computational overhead is the inclusion of electronic polarization effects via charge scaling.^4–6^ The tested recent scaled-charge force field prosECCo75 ^49^ did not induce the non-native salt bridge, had lower occupancies of the two native salt bridges and the dissociation barrier of 1 kcal mol^–1^, which is biologically more realistic than values up to 4–5 kcal mol^–1^ as observed for standard full charge force fields. Such differences have the potential to critically influence computational studies which use MD for evaluating insulin and related peptide structure and their binding toward the receptor.^56–58^

In general, overbinding of charged species in nonpolarizable MD is a known issue which can be addressed within the nonpolarizable framework either via a nonbonded fix or via charge scaling within ECC.^17^ The latter approach, which is physically well justified and therefore preferred also in this study, is currently under active development. The employed scaling factors generally range from 0.75–0.85, with a notion that the former may in some cases slightly underestimate the strength of ion pairing.^16,59^ Other characteristics, such as the charge density or hydrophobicity of the ion, have also bearings on the most appropriate scaling factor.^16^ Finally, new water force fields^60^ compatible with ECC emerge, setting the ground for fully consistent models.

## Computational Methods

### Experimental Structure Selection

Atomistic structural experimental evidence of salt bridges in human insulin and its analogues comes from X-ray crystallography and nuclear magnetic resonance (NMR) spectroscopy. Four high-quality crystal structures with resolution equal or less than 1 Å are available under PDB codes 1MSO,^61^ 3W7Y,^62^ 5E7W,^63^ and 5HQI.^50^ More than a hundred NMR structures are available in the PDB but some are present in hexamers or solved at acidic pH, which affects their structures. We have thus chosen the NMR structural ensemble of the l-His B24 insulin analogue solved at pH = 8 (PDB 2M2N)^46^ The three salt bridges studied are not affected by the Phe-to-His replacement at the B24 position, which points away from them. The presence of the salt bridges in the listed X-ray structures and 30 models of 2M2N was measured as distances between implicated oxygen and nitrogen atoms in PyMol, version 2.3.0.^51^ or VMD, version 1.9.4a57.^64^

### System Setup

The first model from the 30-structure ensemble of l-HisB24 insulin analogue, PDB 2M2N,^46^ was used as the starting structure and prepared in CHARMM-GUI.^65^ The three disulfide bonds (cysteine pairs A6 and A11, A7 and B7, and A20 and B19) were automatically recognized. The A1, A21, B1, and B30 termini were set as charged.

Two standard (ff19SB^47^ and CHARMM36m, C36m^48^) and one charge-scaled (prosECCo75 ^49^) protein force fields were used, as they represent the state-of-the-art in their categories. The partial charge distributions of the charged amino acids forming the salt bridge were presented in . All the protein force fields were combined with TIP3P (the original version^52^ for ff19SB, the modified one^53^ for C36m and prosECCo75) and SPC/E^54^ water models. For simplicity, the original and modified TIP3P water models are referred to throughout the text as TIP3P. We acknowledge that ff19SB may pair well also with the (computationally more demanding) 4-site OPC water model. Nevertheless, to maintain consistency with CHARMM36m, and prosECCo75 water models which were originally parametrized and validated with the TIP3P water model, we opted in this study to use the TIP3P and SPC/E water models in combination with ff19SB as well. The systems were neutralized in a 150 mM NaCl solution using CHARMM-GUI solution builder tool,^65^ with Na^+^ and Cl^–^ modeled using scaled-charge force fields. A cubic unit cell had sides of 5.3 nm.

### MD Details

The MD simulations were carried out using GROMACS 2022.3 and 2022.4 ^66^ and AMBER20 ^67^ software packages for unrestrained MD (MD structural ensembles) and Nuclear Overhauser Enhancement (NOE)-restrained MD (NMR structural ensembles), respectively. We have strived to use the same setup for running the simulations in GROMACS and AMBER but due to different options available, timing of individual steps, or hardware requirements, minor deviations between these protocols occurred as detailed below. These pertained mostly to the lengths of equilibration steps, force constants for positional restraints, thermostat, barostat, or bond-constraining algorithms.

The unrestrained MD simulations were subject to minimization and equilibration in the NVT ensemble for 125 ps with harmonically restrained proteins (force constants of 96 kcal mol^–1^ nm^–2^ for backbones, 10 kcal mol^–1^ nm^–2^ for side chains, and 1 kcal mol^–1^ rad^–2^ for dihedrals). In the NOE-restrained MD, the systems were minimized and equilibrated in NVT and NpT ensembles (60 and 60 ps) with 1 fs time step and harmonically restrained proteins (force constant of 100 kcal mol^–1^ nm^–2^). Both production simulations were propagated with a 2 fs time step at constant temperature (300 K) and constant pressure (1 bar) in NpT ensemble. The unrestrained MD were simulated for 10 μs and employed the Nosé-Hoover thermostat^68–70^ and Parrinello–Rahman barostat,^71^ and all the bonds containing hydrogen were constrained with the LINCS algorithm.^72^ The NOE-restrained MD was simulated for 100 ns and used the Langevin thermostat^73^ and Monte Carlo barostat^74^ and bonds containing hydrogen were constrained with the SHAKE algorithm.^75^ Four replicas of production MD were performed by using the force field combinations mentioned above. The Particle Mesh Ewald (PME) method^76^ was applied to calculate long-range electrostatic interactions. The cutoff distance of 1.2 nm was used for both short-range electrostatic and van der Waals interactions.

To study the dynamical properties of the insulin analogue, the occupancies of all salt bridges in the NMR structural ensemble and our MD trajectories were calculated using MDAnalysis^77,78^ with a cutoff N···O distance of 3.5 Å. The calculated occupancies provide a quantitative measure of the stability and persistence of salt bridge interactions over the simulation time.

Umbrella sampling simulations were conducted to measure the free energy profile for the salt bridge opening, i.e., increasing the C···CZ distance (*r*_C‑CZ_) in A21–B22 and the CD ···N distance (*r*_CD‑N_) in A1–A4. The sampling windows were spaced 0.3 to 0.5 Å apart, and harmonic umbrella potentials with force constants of 72 and 95 kcal mol^–1^ nm^–2^ were applied for N-terminal A1–Glu A4 and C-terminal A21–Arg B22, respectively. The free energy profiles were obtained using the Weighted Histogram Analysis Method (WHAM)^79^ as implemented in GROMACS.^66^ Finally, free energy profiles are corrected by +2*k*_B_ *T*ln­(*r*), which is a volume entropy factor,^80^ and shifted to get the minimum value at zero.

## Supplementary Material





## Data Availability

All input and parameter files necessary to replicate our simulations can be found at https://zenodo.org (DOI: 10.5281/zenodo.15630848).

## Notes

Salt bridges in crystallographic structures (Tables S1–S2), RMSD data of the MD simulations (Figures S1–S2 and Tables S3–S4), salt bridge occurrences using TIP3P and SPC/E (Figure S3 and Tables S5–S7), amino acid partial charges (Figure S4 and Tables S8–S11), and umbrella sampling results using TIP3P and SPC/E (Figures S5–S6) (PDF)
Transparent Peer Review report available (PDF)
