# Supplementary material for: NMR-Derived Salt Bridges in Insulin Analogue: Resolving Artifactual Overbinding in Molecular Dynamics via Charge Scaling
Source: J Phys Chem Lett. 2025 Jul 15;16(29):7436–42. doi: 10.1021/acs.jpclett.5c01786 (PMC12302216; doi:10.1021/acs.jpclett.5c01786)
Supplement: Supplementary file 1 [file jz5c01786_si_001.pdf]

Supplementary Information: NMR-Derived Salt Bridges in Insulin Analog: Resolving Artifactual Overbinding in Molecular Dynamics via Charge Scaling

Ngoc Lan Le Nguyen, Jiří Žák, Pavel Jungwirth, and Martin Lepšík*

Institute of Organic Chemistry and Biochemistry of the Czech Academy of Sciences, Flemingovo nám. 542/2, 160 00 Prague 6, Czech Republic

July 9, 2025

Contents

S1 Experimental Structures S2

S2 Insulin Analog MD Simulations S2

S2.1 Overall Stability S2

S2.2 Salt Bridge Occurrence S4

S2.3 Amino Acid Partial Charges S6

S3 Free Energy Profiles by Umbrella Sampling S10

*martin.lepsik@uochb.cas.cz

S1 Experimental Structures

Salt bridges in crystallographic X-ray structures of human insulin and insulin analog.

Table S1: The distances of N···O in the three salt bridges obtained from the crystallographic X-ray structures of human insulin and insulin analog

Atom pairs	Distance of the atom pairs (Å)								
	1MSO(1)	1MSO(2)	3W7Y(1)	3W7Y(2)	5E7W(1)A	5E7W(1)B	5E7W(2)	5HQI(1)A	5HQI(1)B
**N-terminal A1–Glu A4**									
**N···OE1**	2.8	2.8	2.8	4.9	2.8	2.8	2.7	2.9	2.9
**N···OE2**	5.0	5.0	4.9	2.7	5.0	5.0	4.8	5.1	5.1
**C-terminal A21–Arg B22**									
**NE···O**	5.2	3.0	3.0	3.2	5.0	5.5	3.1	5.0	2.8
**NH1···O**	5.7	4.7	4.7	3.3	5.7	5.9	3.1	6.9	2.8
**NH2···O**	7.0	3.0	2.9	4.8	6.9	7.3	4.7	5.2	4.5
**NE···OXT**	7.3	5.0	4.8	5.1	7.2	7.2	5.1	7.1	4.7
**NH1···OXT**	7.7	6.7	6.7	5.0	7.6	7.9	4.9	8.9	4.2
**NH2···OXT**	9.1	4.8	4.9	6.8	9.0	9.2	6.8	7.1	6.3
**Glu A17–Arg B22**									
**NE···OE1**	7.0	7.7	7.7	7.9	7.0	7.0	7.6	6.6	7.4
**NH1···OE1**	5.1	5.5	5.5	7.4	5.0	5.0	7.2	4.8	6.8
**NH2···OE1**	5.3	7.3	7.1	5.6	5.3	5.3	5.4	4.6	5.1
**NE···OE2**	5.0	6.1	6.0	6.1	4.9	4.9	6.1	5.3	6.9
**NH1···OE2**	3.1	4.0	3.8	6.2	3.0	3.0	6.2	3.2	6.9
**NH2···OE2**	3.4	6.2	5.8	4.1	3.3	3.3	4.1	4.0	4.8

Table S2: The presence of salt bridges in crystallographic X-ray structures of human insulin and insulin analog by comparing their N···O distances with the cutoff of 3.5 Å.

Salt bridge	Salt bridge occurence (1: YES; 0: NO)							
	1MSO(1)	1MSO(2)	3W7Y(1)	3W7Y(2)	5E7W(1)	5E7W(2)	5HQI(1)A	5HQI(1)B
**N-terminal A1–Glu A4**	**1**	**1**	**1**	**1**	**1**	**1**	**1**	**1**
**C-terminal A21–Arg B22**	**0**	**1**	**1**	**1**	**0**	**1**	**0**	**1**
**Glu A17–Arg B22**	**1**	**0**	**0**	**0**	**1**	**0**	**1**	**0**

S2 Insulin Analog MD Simulations

S2.1 Overall Stability

The RMSD data and average RMSD values for the secondary structure backbone of the insulin analog across four replicas of NOE-restrained and unrestrained MD simulations, using the six force fields: ff19SB TIP3P, C36m TIP3P, prosECCo75 TIP3P, ff19SB SPC/E, C36m SPC/E, and prosECCo75 SPC/E are presented in Tables S3, S4 and Figures S1, S2.

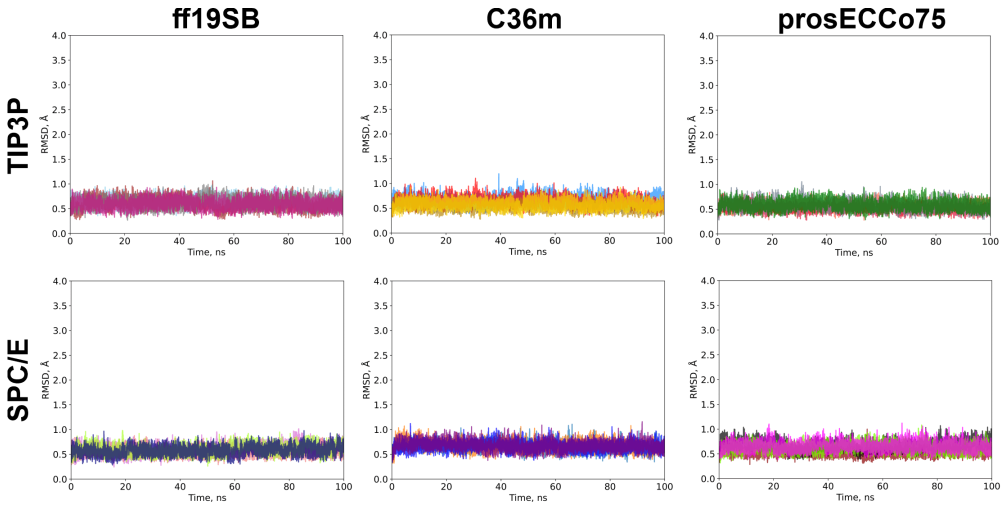

Figure S1: Secondary structure backbone root-mean-square deviation (RMSD) of insulin analog obtained from the four NOE-restrained MD replicas (100 ns) for each force field of ff19SB TIP3P, C36m TIP3P, prosECCo75 TIP3P, ff19SB SPC/E, C36m SPC/E, and prosECCo75 SPC/E.

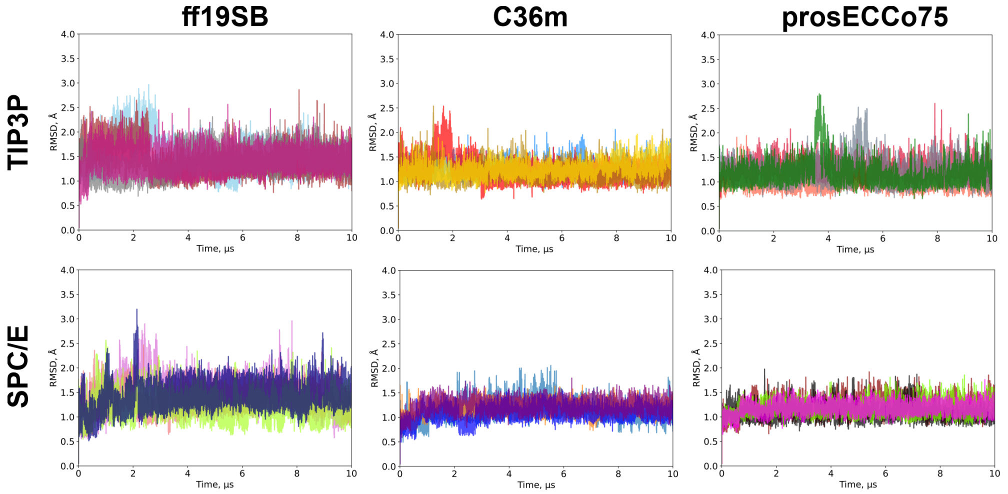

Figure S2: Secondary structure backbone root-mean-square deviation (RMSD) of insulin analog obtained from the four unrestrained MD replicas (10 μs) for each force field of ff19SB TIP3P, C36m TIP3P, prosECCo75 TIP3P, ff19SB SPC/E, C36m SPC/E, and prosECCo75 SPC/E.

Table S3: Average RMSD and its standard deviation (SD) data of secondary structure backbone of the insulin analog in NOE-restraints MD simulations using the six force fields

	Force fields	Average RMSD (Å)	SD (Å)
1	ff19SB TIP3P	0.60	0.017
2	C36m TIP3P	0.60	0.055
3	prosECCo75 TIP3P	0.57	0.003
4	ff19SB SPC/E	0.58	0.020
5	C36m SPC/E	0.67	0.009
6	prosECCo75 SPC/E	0.62	0.056

Table S4: Average RMSD and its standard deviation (SD) data of secondary structure backbone of the insulin analog in unrestrained MD simulations using the six force fields

	Force fields	Average RMSD (Å)	SD (Å)
1	ff19SB TIP3P	1.42	0.245
2	C36m TIP3P	1.22	0.195
3	prosECCo75 TIP3P	1.18	0.207
4	ff19SB SPC/E	1.35	0.259
5	C36m SPC/E	1.16	0.169
6	prosECCo75 SPC/E	1.16	0.155

S2.2 Salt Bridge Occurrence

The salt bridge occupancies of N-terminal A1–Glu A4, C-terminal A21–Arg B22, and Glu A17–Arg B22 from NOE-restraints and unrestrained MD simulations are presented in Tables S5–S7 and Figure S3.

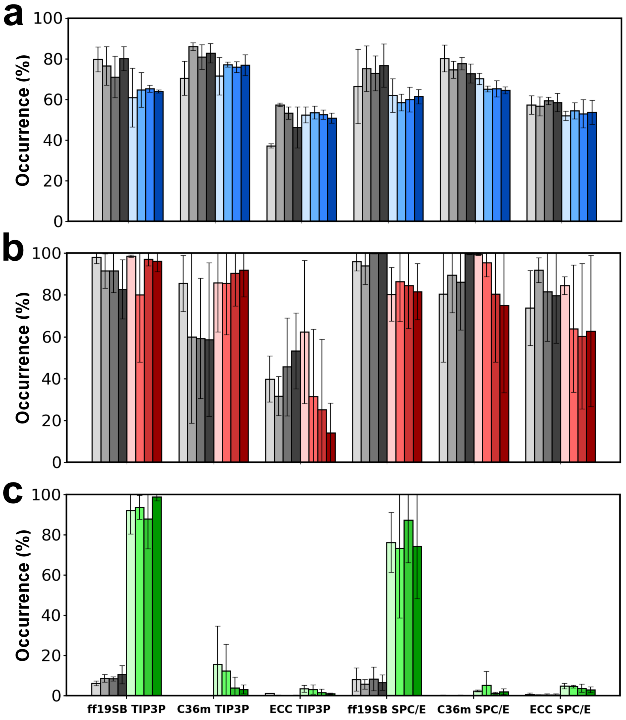

Figure S3: Salt bridge occurrences in MD simulations using TIP3P and SPC/E water models. NOE-restrained MDs are in gray and unrestrained MDs are colored blue, red, and green, respectively, for a) A1–A4, b) A21–B22, and c) A17–B22. Note that the NOE-restrained occurrences are non-existent for C36m and prosECCo75 for A17–B22. Each trajectory was divided into four segments of equal length. The height of the bars are averages of the four replicas and the error bars are the standard deviations.

Table S5: Occurrence of N-terminal A1–Glu A4 in the NOE-restrained and unrestrained MDs using the six force fields

N-terminal A1–Glu A4

Simulation	ff19SB TIP3P							ff19SB SPC/E						
	NOE-restrained				Unrestrained			NOE-restrained				Unrestrained		
**Time segment**	**1**	**2**	**3**	**4**	**2**	**3**	**4**	**1**	**2**	**3**	**4**	**2**	**3**	**4**
**Occurrence (%)**	80	76	71	80	65	65	64	67	75	73	77	58	60	61
**SD (%)**	6	10	10	6	9	2	1	18	11	9	11	4	6	3
**Simulation**	**C36m TIP3P**							**C36m SPC/E**						
	**NOE-restrained**				**Unrestrained**			**NOE-restrained**				**Unrestrained**		
**Time segment**	**1**	**2**	**3**	**4**	**2**	**3**	**4**	**1**	**2**	**3**	**4**	**2**	**3**	**4**
**Occurrence (%)**	70	86	81	83	77	76	77	80	75	78	73	65	65	65
**SD (%)**	8	2	6	5	1	3	5	6	4	3	5	1	4	2
**Simulation**	**prosECCo75 TIP3P**							**prosECCo75 SPC/E**						
	**NOE-restrained**				**Unrestrained**			**NOE-restrained**				**Unrestrained**		
**Time segment**	**1**	**2**	**3**	**4**	**2**	**3**	**4**	**1**	**2**	**3**	**4**	**2**	**3**	**4**
**Occurrence (%)**	37	57	53	46	54	53	51	57	57	59	58	54	53	54
**SD (%)**	1	1	3	10	3	2	2	5	5	2	5	4	7	6

Table S6: Occurrence of C-terminal A21–Arg B22 in the NOE-restrained and unrestrained MDs using the six force fields

C-terminal A21–Arg B22

Simulation	ff19SB TIP3P							ff19SB SPC/E						
	NOE-restrained				Unrestrained			NOE-restrained				Unrestrained		
**Time segment**	**1**	**2**	**3**	**4**	**2**	**3**	**4**	**1**	**2**	**3**	**4**	**2**	**3**	**4**
**Occurrence (%)**	98	91	92	83	80	97	96	96	94	100	100	86	85	82
**SD (%)**	3	8	10	14	32	3	5	4	9	0	0	19	21	13
**Simulation**	**C36m TIP3P**							**C36m SPC/E**						
	**NOE-restrained**				**Unrestrained**			**NOE-restrained**				**Unrestrained**		
**Time segment**	**1**	**2**	**3**	**4**	**2**	**3**	**4**	**1**	**2**	**3**	**4**	**2**	**3**	**4**
**Occurrence (%)**	86	60	59	59	86	90	92	80	89	86	100	95	80	75
**SD (%)**	13	41	29	37	24	16	13	32	18	23	0	7	33	42
**Simulation**	**prosECCo75 TIP3P**							**prosECCo75 SPC/E**						
	**NOE-restrained**				**Unrestrained**			**NOE-restrained**				**Unrestrained**		
**Time segment**	**1**	**2**	**3**	**4**	**2**	**3**	**4**	**1**	**2**	**3**	**4**	**2**	**3**	**4**
**Occurrence (%)**	40	32	46	53	31	25	14	74	92	82	80	64	60	63
**SD (%)**	11	9	23	18	32	34	14	18	6	24	23	30	35	36

Table S7: Occurrence of Glu A17–Arg B22 in in the NOE-restrained and unrestrained MDs using the six force fields

Glu A17–Arg B22

Simulation	ff19SB TIP3P							ff19SB SPC/E						
	NOE-restrained				Unrestrained			NOE-restrained				Unrestrained		
**Time segment**	**1**	**2**	**3**	**4**	**2**	**3**	**4**	**1**	**2**	**3**	**4**	**2**	**3**	**4**
**Occurrence (%)**	6	9	8	10	94	88	99	8	6	8	6	73	87	74
**SD (%)**	1	2	1	5	6	15	2	6	2	6	4	34	21	26
**Simulation**	**C36m TIP3P**							**C36m SPC/E**						
	**NOE-restrained**				**Unrestrained**			**NOE-restrained**				**Unrestrained**		
**Time segment**	**1**	**2**	**3**	**4**	**2**	**3**	**4**	**1**	**2**	**3**	**4**	**2**	**3**	**4**
**Occurrence (%)**	0	0	0	0	12	4	3	0	0	0	0	5	1	2
**SD (%)**	0	0	0	0	13	5	2	0	0	0	0	7	1	1
**Simulation**	**prosECCo75 TIP3P**							**prosECCo75 SPC/E**						
	**NOE-restrained**				**Unrestrained**			**NOE-restrained**				**Unrestrained**		
**Time segment**	**1**	**2**	**3**	**4**	**2**	**3**	**4**	**1**	**2**	**3**	**4**	**2**	**3**	**4**
**Occurrence (%)**	1	0	0	0	3	1	1	1	0	0	0	4	4	3
**SD (%)**	0	0	0	0	2	2	0	1	0	0	0	1	2	1

S2.3 Amino Acid Partial Charges

Figure S4 and Tables S8–S11 report the atomic partial charges of the amino acid residues participating in the investigated salt bridges.

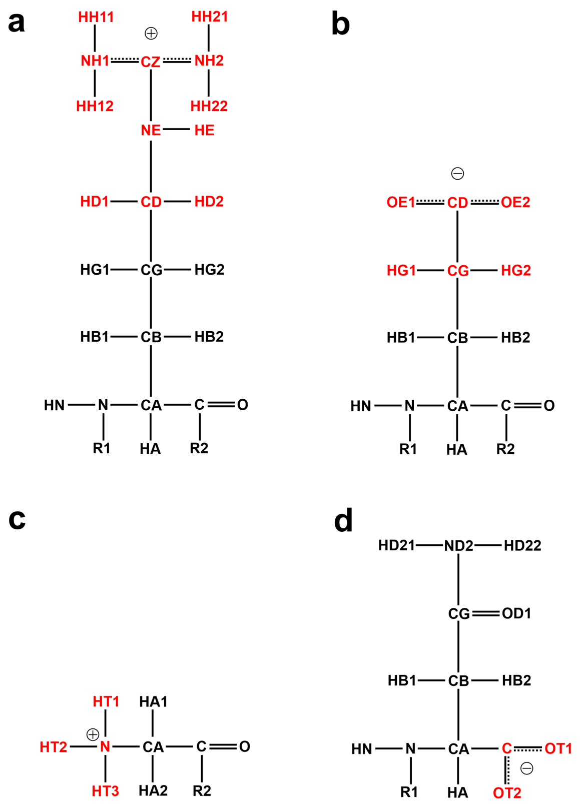

Figure S4: Atomic structures and partial charges of the amino acid residues and termini in the salt bridges of the analog. a) Positively charged arginine (ARG) side chain, b) Negatively charged glutamic acid (GLU) side chain, c) Positively charged glycine (GLY) N-terminal, d) Negatively charged asparagine (ASN) C-terminal. Atoms bearing formal charges are labeled by the atom names (C36m force field), in which the charge-scaled atoms are highlighted in red.

Table S8: Atomic partial charges of ARG(+1) in ff19SB, C36m, and prosECCo75 force fields. Charge-scaled parts are highlighted in red.

**Atom Name**		**Atom Type**		**Atomic Partial Charges**		
**ff19SB**	**C36m /prosECCo75**	**ff19SB**	**C36m /prosECCo75**	**ff19SB**	**C36m**	**prosECCo75**
N	N	N	NH1	-0.3497	-0.47	-0.4700
H	HN	H	H	0.2747	0.31	0.3100
CA	CA	XC9	CT1	-0.2637	0.07	0.0700
HA	HA	H1	HB1	0.1560	0.09	0.0900
CB	CB	C8	CT2	-0.0007	-0.18	-0.1800
HB2	HB1	HC	HA2	0.0327	0.09	0.0900
HB3	HB2	HC	HA2	0.0327	0.09	0.0900
CG	CG	C8	CT2	0.0390	-0.18	-0.1800
HG2	HG1	HC	HA2	0.0285	0.09	0.0900
HG3	HG2	HC	HA2	0.0285	0.09	0.0900
CD	CD	C8	CT2	0.0486	0.20	0.1500
HD2	HD1	H1	HA2	0.0687	0.09	0.0675
HD3	HD2	H1	HA2	0.0687	0.09	0.0675
NE	NE	N2	NC2	-0.5295	-0.70	-0.5250
HE	HE	H	HC	0.3456	0.44	0.3300
CZ	CZ	CA	C	0.8076	0.64	0.4800
NH1	NH1	N2	NC2	-0.8627	-0.80	-0.6000
HH11	HH11	H	HC	0.4478	0.46	0.3450
HH12	HH12	H	HC	0.4478	0.46	0.3450
NH2	NH2	N2	NC2	-0.8627	-0.80	-0.6000
HH21	HH21	H	HC	0.4478	0.46	0.3450
HH22	HH22	H	HC	0.4478	0.46	0.3450
C	C	C	C	0.7341	0.51	0.5100
O	O	O	O	-0.5894	-0.51	-0.5100
			**Total**	**1.0000**	**1.00**	**0.7500**

Table S9: Atomic partial charges of GLU(-1) in ff19SB, C36m, and prosECCo75 force fields. Charge-scaled parts are highlighted in red.

**Atom Name**		**Atom Type**		**Atom Partial Charges**		
**ff19SB**	**C36m /prosECCo75**	**ff19SB**	**C36m /prosECCo75**	**ff19SB**	**C36m**	**prosECCo75**
N	N	N	NH1	-0.5163	-0.47	-0.4700
H	HN	H	H	0.2936	0.31	0.3100
CA	CA	XC1	CT1	0.0397	0.07	0.0700
HA	HA	H1	HB1	0.1105	0.09	0.0900
CB	CB	2C	CT2A	0.0560	-0.18	-0.1800
HB2	HB1	HC	HA2	-0.0173	0.09	0.0900
HB3	HB2	HC	HA2	-0.0173	0.09	0.0900
CG	CG	2C	CT2	0.0136	-0.28	-0.2100
HG2	HG1	HC	HA2	-0.0425	0.09	0.0675
HG3	HG2	HC	HA2	-0.0425	0.09	0.0675
CD	CD	CO	CC	0.8054	0.62	0.4650
OE1	OE1	O2	OC	-0.8188	-0.76	-0.5700
OE2	OE2	O2	OC	-0.8188	-0.76	-0.5700
C	C	C	C	0.5366	0.51	0.5100
O	O	O	O	-0.5819	-0.51	-0.5100
			**Total**	**-1.0000**	**-1.00**	**-0.7500**

Table S10: Atomic partial charges of GLY N-term(+1) in ff19SB, C36m, and prosECCo75 force fields. Charge-scaled parts are highlighted in red.

**Atom Name**		**Atom Type**		**Atomic Partial Charges**		
**ff19SB**	**C36m /prosECCo75**	**ff19SB**	**C36m /prosECCo75**	**ff19SB**	**C36m**	**prosECCo75**
N	N	N3	NH3	0.2943	-0.30	-0.3025
H1	HT1	H	HC	0.1642	0.33	0.2475
H2	HT2	H	HC	0.1642	0.33	0.2475
H3	HT3	H	HC	0.1642	0.33	0.2475
CA	CA	CX	CT2	-0.0100	0.13	0.1300
HA2	HA1	HP	HB2	0.0895	0.09	0.0900
HA3	HA2	HP	HB2	0.0895	0.09	0.0900
C	C	C	C	0.6163	0.51	0.5100
O	O	O	O	-0.5722	-0.51	-0.5100
			**Total**	**1.0000**	**1.00**	**0.7500**

Table S11: Atomic partial charges of ASN C-term(-1) in ff19SB, C36m, and prosECCo75 force fields

**Atom Name**		**Atom Type**		**Atomic Partial Charges**		
**ff19SB**	**C36m /prosECCo75**	**ff19SB**	**C36m /prosECCo75**	**ff19SB**	**C36m**	**prosECCo75**
N	N	N	NH1	-0.3821	-0.47	-0.47
H	HN	H	H	0.2681	0.31	0.31
CA	CA	CX	CT1	-0.2080	0.07	0.07
HA	HA	H1	HB1	0.1358	0.09	0.09
CB	CB	2C	CT2	-0.2299	-0.18	-0.18
HB2	HB1	HC	HA2	0.1023	0.09	0.09
HB3	HB2	HC	HA2	0.1023	0.09	0.09
CG	CG	C	CC	0.7153	0.55	0.55
OD1	OD1	O	O	-0.6010	-0.55	-0.55
ND2	ND2	N	NH2	-0.9084	-0.62	-0.62
HD21	HD21	H	H	0.4150	0.32	0.32
HD22	HD22	H	H	0.4150	0.30	0.30
C	C	C	CC	0.8050	0.34	0.39
O	OT1	O2	OC	-0.8147	-0.67	-0.57
OXT	OT2	O2	OC	-0.8147	-0.67	-0.57
			**Total**	**-1.0000**	**-1.00**	**-0.75**

S3 Free Energy Profiles by Umbrella Sampling

Figure S5 illustrates the PMF profiles of salt bridge opening of C-terminal A21–Arg B22 and N-terminal A1–Glu A4 obtained from the umbrella sampling simulations using the six force fields of ff19SB TIP3P, C36m TIP3P, prosECCo75 TIP3P, ff19SB SPC/E, C36m SPC/E, and prosECCo75 SPC/E. Moreover, the estimated boundaries of direct contacts and solvent-shared configurations of these salt bridges are demonstrated in Figure S6.

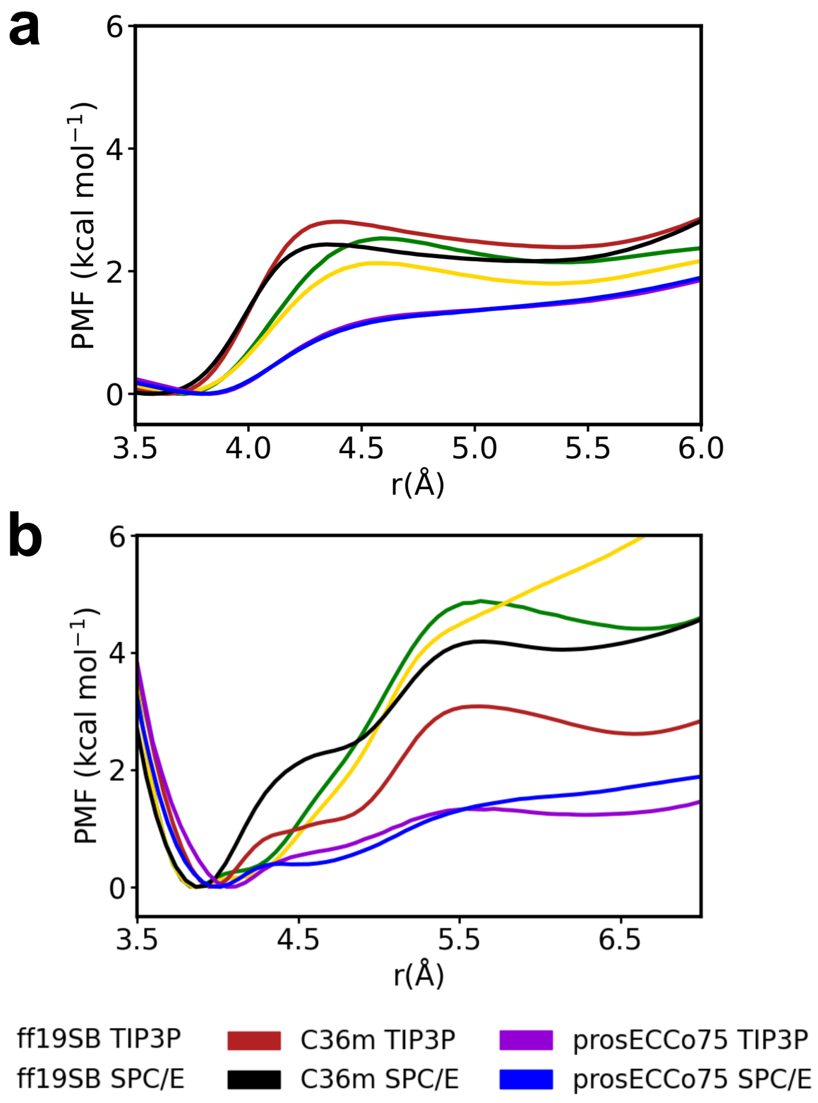

Figure S5: Potential of mean force (PMF) results obtained from the umbrella sampling simulations using the six force field setups a) N-terminal A1–Glu A4. b) C-terminal A21–Arg B22

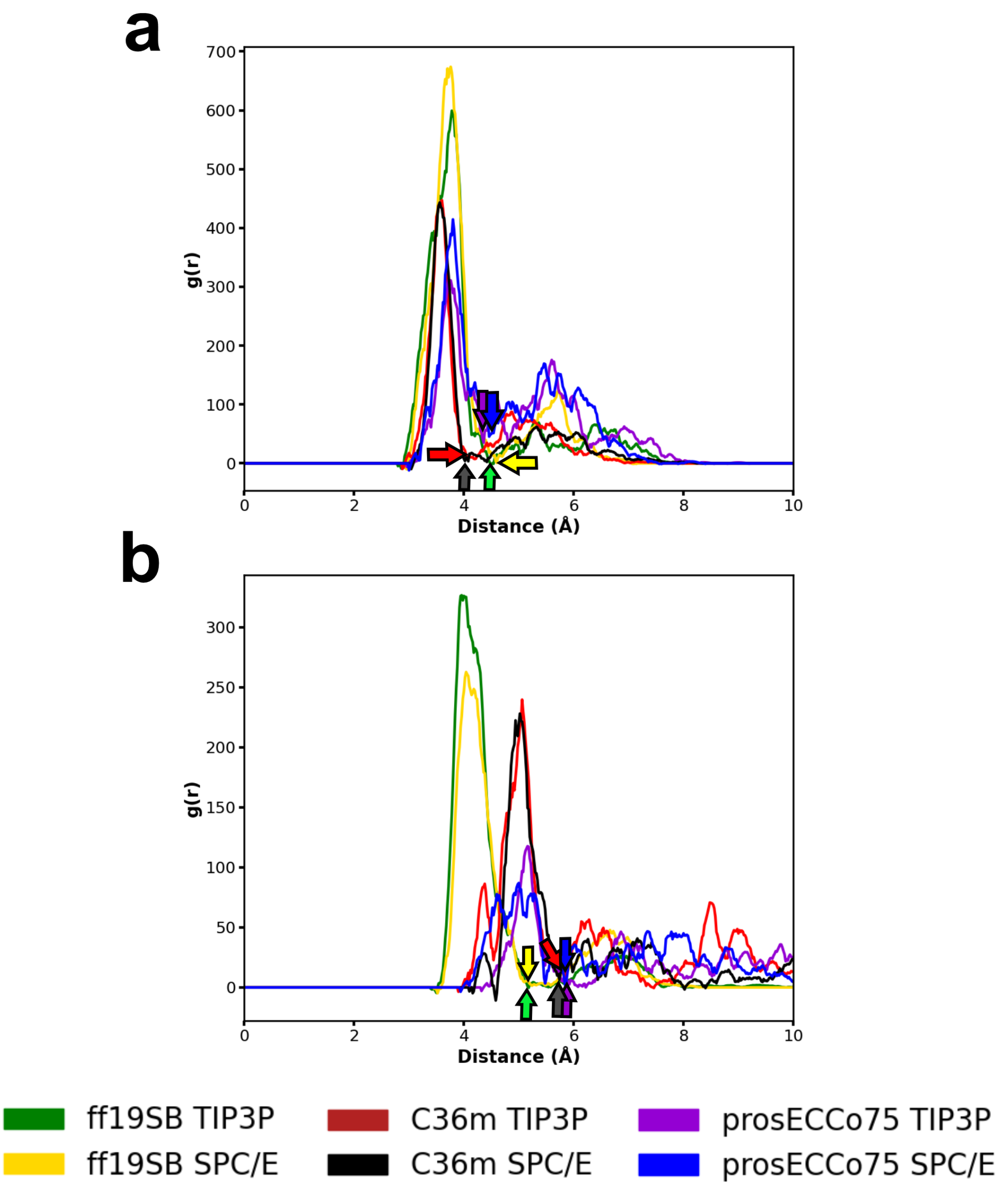

Figure S6: Radial distribution functions (RDF) of a) CD–N atoms in N-terminal A1–Glu A4 and b) C–CZ atoms in C-terminal A21–Arg B22 obtained from the 500-ps pulling simulations which were performed to generate initial configurations for the umbrella sampling windows. The arrows at the first minimum regions indicate the estimated boundary of the direct contacts and solvent-shared configurations.
