# Supplementary material for: NMR-Derived Salt Bridges in Insulin Analogue: Resolving Artifactual Overbinding in Molecular Dynamics via Charge Scaling
Source: J Phys Chem Lett. 2025 Jul 15;16(29):7436–42. doi: 10.1021/acs.jpclett.5c01786 (PMC12302216; doi:10.1021/acs.jpclett.5c01786)
Supplement: Supplementary file 2 [file jz5c01786_si_002.pdf]

jz-2025-01786f.R1

Name: Peer Review Information for "NMR-Derived Salt Bridges in Insulin Analog: Resolving Artifactual Overbinding in Molecular Dynamics via Charge Scaling"

First Round of Reviewer Comments

Reviewer: 1

Comments to the Author

The manuscript brings clear evidence of the overbinding caused by the standard and commonly used molecular force field vs. the superior performance of the recent prosECCo75 force field applying scaled charges. The results are well documented by extensive simulation evidence. I appreciate the combination of NOE-restrained and unrestrained simulations, accompanied by extensive free energy calculations. The combination of these approaches allows to clearly identify the measure of overbinding.

The manuscript is accompanied by very detailed Supporting information + 8.2 GB Zenodo archive with simulation input and output files. The level of sharing the data is above the standards.

The relevance of the conclusions of this manuscript (which is not limited to the study of the investigated molecules) to biomolecular simulations is well formulated.

I recommend accepting the manuscript nearly as it is, addressing only minor points noted below. The manuscript does not need to be reviewed again.

1) Page 1, lines 42-44, right column: The sentence “The results show that salt bridge strength decreases with increasing hydration—from approximately 2.8 kcal mol−1 in bulk water to around 2.1 kcal mol−1 in a 100-water molecule cluster...” does not make sense to me – bulk water represents higher hydration…

2) Page 2, lines 12-13, left column: The sentence “These peptides are comprise two interconnected chains,...” requires language improvement.

3) Page 3, lines 19-26, left column: The authors might consider merging the sentence “Consistently, unrestrained…” with the preceding paragraph formed by a single sentence.

4) Page 3, line 50, left column: The sentence “each other Figure 3, upper left panel)” lacks “(“.

5) Page 4, line 18, right column: The text “Åare” lacks a space.

6) Supplementary information, Page S11: “500-ps pulling simulation” - not clear, which pulling simulation is meant.

7) Supplementary information, Page S11: “The arrows the first minima indicate” – this sentence requires language improvement.

Reviewer: 2

Comments to the Author

In this manuscript, the authors investigated the problem inherent to non-polarizable force fields: tendency to overestimate the strength of salt bridges between charged residues. Previous MD simulation studies have primarily focused on the stability of salt bridges in simplified systems such as amino acid analogs or capped dipeptides. Here, the authors employed all-atom MD simulations (restrained and unrestrained) and umbrella sampling calculations to establish and quantify the overbinding effect of salt bridges in several common non-polarizable protein force fields. The authors combined protein force fields with several water models (TIP3P and SPC/E) while using various insulin peptide structures as their model system. As an important finding of the manuscript, the authors show that the overbinding effect of salt bridging interactions in these relatively large systems has a high value of up to 4-5 kcal/mol for CHARMM36m and ff19SB, two popular protein force fields in the simulation community. The authors further show that prosECCo75 better captures salt bridging interactions providing a more realistic stabilization of salt bridges than CHARMM36m and ff19SB.

Overall, the manuscript shows overstabilization of salt bridges in two popular protein molecular mechanics force fields on a reliable model system which exceeds previously reported examples of systems with salt bridges. The authors further propose how the overbinding can be decreased within the non-polarizable force field framework. The manuscript is a short technical article with important findings about commonly used molecular mechanics force fields and salt bridges. The manuscript is publishable in The Journal of Physical Chemistry Letters after minor revisions.

Minor technical suggestions and clarifications:

1. Salt bridges are defined as interactions between charged amino acid side chains, but can the authors also provide additional criteria for the formation of a salt bridge, for example if a distance cutoff was used?

2. The authors mention that water dynamics and solvent exposure affects the stability of a salt bridge – can the authors clarify the rationale behind choosing TIP3P and SPC/E water models? For example, in J. Chem. Theory Comput. 2020, 16, 1, 528–552 the authors suggest the usage of the OPC water model with ff19SB protein force field rather than TIP3P.

3. Page 4, lines 54-55: "The same parameters were used as often as possible." Can the authors clarify what is meant by the same parameters? Are these parameters from force field, NOE-restraints or anything else?

Minor errors to fix:

Page 2, line 12: "These peptides are comprise two interconnected..." – should be "are comprised of".

Page 4, line 18: "crystal structures with resolution equal or less than 1 Åare" – Å and are written together.

Page 4, line 42: "TIP3P (the original version for ff19SB," – a bracket is missing.

Author's Response to Peer Review Comments:

Dr. Martin Lepšík, Ph.D.
senior researcher
Computational Chemistry for
Drug Design
IOCB Prague
Czech Republic
E-mail: lepsik@uochb.cas.cz

Prague, July 9, 2025

Dear Editor,

We are submitting the revised version of our manuscript entitled “NMR-Derived Salt Bridges in Insulin Analog: Resolving Artifactual Overbinding in Molecular Dynamics via Charge Scaling”.

We are very grateful to the reviewers for their positive appreciation of our manuscript, their careful reading, and their relevant suggestions and questions, which helped us improve it. Following their suggestions, we clarified the text in several points. We have also checked all the technical issues reported by the editorial office.

You will find below our detailed answers to the reviewers and responses to the technical issues, together with the list of changes that were made accordingly. We believe that this revised version addresses all the comments formulated by the reviewers.

Sincerely,
Martin Lepšík

REVIEWER(S)’ REPORT(S):
Author answers
Added text

Reviewer(s)' Comments to Author:

Reviewer: 1

Comments:

The manuscript brings clear evidence of the overbinding caused by the standard and commonly used molecular force field vs. the superior performance of the recent prosECCo75 force field applying scaled charges. The results are well documented by extensive simulation evidence. I appreciate the combination of NOE-restrained and unrestrained simulations, accompanied by

extensive free energy calculations. The combination of these approaches allows to clearly identify the measure of overbinding.
The manuscript is accompanied by very detailed Supporting information + 8.2 GB Zenodo archive with simulation input and output files. The level of sharing the data is above the standards.
The relevance of the conclusions of this manuscript (which is not limited to the study of the investigated molecules) to biomolecular simulations is well formulated.

I recommend accepting the manuscript nearly as it is, addressing only minor points noted below. The manuscript does not need to be reviewed again.

We thank the reviewer for their kind appreciation and encouraging feedback. We are pleased that the reviewer finds our results well supported by extensive simulation data and appreciates the combined use of NOE-restrained and unrestrained simulations alongside the free energy calculations. We also thank the reviewer for highlighting our efforts in data sharing and the broader relevance of our conclusions to the field of biomolecular simulations. We are grateful for the recommendation to accept the manuscript with only minor revisions, and we have carefully addressed the specific points raised in the text below.

1) Page 1, lines 42-44, right column: The sentence "The results show that salt bridge strength decreases with increasing hydration—from approximately 2.8 kcal mol−1 in bulk water to around 2.1 kcal mol−1 in a 100-water molecule cluster…" does not make sense to me – bulk water represents higher hydration…

We thank the reviewer for spotting our error in interpreting the literature data. We now correct the text including the free energies according to the cited article. The revised text now reads: *"The results show that salt bridge strength decreases with increasing hydration---from approximately 3.7 kcal mol $^{-1}$ in a 20-water molecule cluster to around 2.2 kcal mol $^{-1}$ in a 150water molecule cluster - highlighting the role of solvent screening in modulating electrostatic interactions. To extrapolate to the bulk solvent, a smaller model, namely the NH_4^+...$HCOO^-$ ion pair, was calculated, yielding nearly identical results (approximately 3.9 kcal mol $^{-1}$ in a 20-water molecule cluster and 2.4 kcal mol $^{-1}$ in the bulk water)."*

2) Page 2, lines 12-13, left column: The sentence "These peptides are comprise two interconnected chains,…" requires language improvement.

We thank the reviewer for pointing this out and have now improved the language. The corrected sentence now reads:
"These peptides consist of two interconnected chains, A and B, which make up in total about 50 amino acids."

3) Page 3, lines 19-26, left column: The authors might consider merging the sentence "Consistently, unrestrained..." with the preceding paragraph formed by a single sentence.

This is a helpful suggestion. We have merged the sentence with the preceding paragraph.

4) Page 3, line 50, left column: The sentence "each other Figure 3, upper left panel)" lacks "(".

We thank the reviewer for catching this oversight. The missing opening parenthesis was added.

5) Page 4, line 18, right column: The text "Åare" lacks a space.

The text was edited.

6) Supplementary information, Page S11: "500-ps pulling simulation" - not clear, which pulling simulation is meant.

We thank the reviewer for pointing out the ambiguity. We have clarified the description to specify that the "500-ps pulling simulation" refers to the steered molecular dynamics (SMD) simulation used to generate the initial configurations for umbrella sampling. The revised text now reads:

"500-ps pulling simulations which were performed to generate initial configurations for the umbrella sampling windows"

7) Supplementary information, Page S11: "The arrows the first minima indicate" – this sentence requires language improvement.

We have modified the sentence. The whole revised sentence now reads:

"The arrows at the first minimum regions indicate the estimated boundary of the direct contacts and solvent-shared configurations."

Reviewer: 2

Comments:
In this manuscript, the authors investigated the problem inherent to non-polarizable force fields: tendency to overestimate the strength of salt bridges between charged residues. Previous MD simulation studies have primarily focused on the stability of salt bridges in simplified systems such as amino acid analogs or capped dipeptides. Here, the authors employed all-atom MD simulations (restrained and unrestrained) and umbrella sampling calculations to establish and quantify the

overbinding effect of salt bridges in several common non-polarizable protein force fields. The authors combined protein force fields with several water models (TIP3P and SPC/E) while using various insulin peptide structures as their model system. As an important finding of the manuscript, the authors show that the overbinding effect of salt bridging interactions in these relatively large systems has a high value of up to 4-5 kcal/mol for CHARMM36m and ff19SB, two popular protein force fields in the simulation community. The authors further show that prosECCo75 better captures salt bridging interactions providing a more realistic stabilization of salt bridges than CHARMM36m and ff19SB.

Overall, the manuscript shows overstabilization of salt bridges in two popular protein molecular mechanics force fields on a reliable model system which exceeds previously reported examples of systems with salt bridges. The authors further propose how the overbinding can be decreased within the non-polarizable force field framework. The manuscript is a short technical article with important findings about commonly used molecular mechanics force fields and salt bridges. The manuscript is publishable in The Journal of Physical Chemistry Letters after minor revisions.

We greatly appreciate the reviewer's thoughtful and positive assessment of our manuscript. We are glad that the significance of our work on salt bridge overbinding in commonly used force fields, as well as the advantages of prosECCo75, have been recognized. Thank you for your constructive feedback and for recommending the manuscript for publication pending minor revisions. We have thoroughly addressed the reviewer's suggestions to improve the manuscript.

Minor technical suggestions and clarifications:

1. Salt bridges are defined as interactions between charged amino acid side chains, but can the authors also provide additional criteria for the formation of a salt bridge, for example if a distance cutoff was used?

The cutoff distance of 3.5 Å was added to the first paragraph on page 2.

2. The authors mention that water dynamics and solvent exposure affects the stability of a salt bridge – can the authors clarify the rationale behind choosing TIP3P and SPC/E water models? For example, in J. Chem. Theory Comput. 2020, 16, 1, 528–552 the authors suggest the usage of the OPC water model with ff19SB protein force field rather than TIP3P.

We thank the reviewer for raising this important point. We agree that a note on this choice is due and thus have added this text to the "Computational Methods" section:

"We acknowledge that ff19SB may pair well also with the (computationally more demanding) 4-site OPC water model. Nevertheless, to maintain consistency with CHARMM36m, and prosECCo75 water models which were originally parameterized and validated with the TIP3P water model, we opted in this study to use the TIP3P and SPC/E water models in combination with ff19SB as well."

3. Page 4, lines 54-55: “The same parameters were used as often as possible.” Can the authors clarify what is meant by the same parameters? Are these parameters from force field, NOErestraints or anything else?

We agree with the reviewer that this sentence was unclear. Therefore, we replace it with the following text:

“We have strived to use the same setup for running the simulations in GROMACS and AMBER but due to different options available, timing of individual steps, or hardware requirements, minor deviations between these protocols occurred as detailed below. These pertained mostly to the lengths of equilibration steps, force constants for positional restraints, thermostat, barostat or bond-constraining algorithms.“

Minor errors to fix:

Page 2, line 12: “These peptides are comprise two interconnected…” – should be “are comprised of”.

We thank the reviewer for pointing this out and have now improved the language. The corrected sentence now reads:

“These peptides consist of two interconnected chains, A and B, which make up in total about 50 amino acids.”

Page 4, line 18: “crystal structures with resolution equal or less than 1 Åare” – Å and are written together.

The text was corrected.

Page 4, line 42: “TIP3P (the original version for ff19SB,” – a bracket is missing. The text was corrected.

TECHNICAL ISSUES:
Author answers

Funding Sources: Authors are required to report ALL funding sources and grant/award numbers relevant to this manuscript. Confirm all sources of funding for ALL authors relevant to this manuscript are included in BOTH the submission form and in the manuscript file to meet this requirement.

Checked.

1. An Abstract, which should summarize the reason for the work, the most significant results, and the conclusions, must be present and labeled.

Checked.

2. Please provide a brief, nonsentence description of the actual contents of each Supporting Information file.

The Supporting Information section in the revised main text has been edited.

3. The TOC graphic should fit in an area no larger than 3.25 in. × 1.75 in. (approx. 8.25 cm × 4.45 cm) and should have adequate resolution and clarity. Confirm that all text is legible at this size.

Checked.
